# Half-Yearly Report of the Physician to the Hospital for Small-Pox and Vaccination, December 19, 1822

**Published:** 1823-02

**Authors:** George Gregory

**Affiliations:** Physician to the Small-Pox and Vaccination Hospital. 8, Upper John-street, Golden-square


### Art. II.—*Half-yearly Report of the Physician to the Hospital for Small-Pox and Vaccination, December* 19, 1822.

The extent to which small-pox has prevailed in London during the last six months, and the consequent increase in the number of persons admitted to the benefits of your Institution, demand, in the first instance, the especial notice of your physician. The admissions during the current year are, with the exception of 1805, the most numerous which the records of the hospital present since the commencement of the present century, and they are not very far distant from the average number of admissions in the earlier periods of the Institution. This fact is, of itself, a sufficient proof that the changes which have taken place in the world during the last twenty years, in reference to the small-pox, have not removed the necessity for an Institution for the express relief of that disease; and that, in point of fact, such an establishment still possesses the most powerful and unquestionable claims upon the support of the public.

From the date of your physician's last report (June 6, 1822,) to the present time, 184 patients have been received, labouring under the casual small-pox: of these, 32 have died, 70 have been discharged cured, and 13 remain in the hospital. The total number of admissions and discharges, therefore, for the present year, stands as follows:—

| 1822. | | *Of whom there* Died. | Were discharg-ed cured. | Remain in Hospital. | Total. |
|---|---|---|---|---|---|
| Admitted from Jan. 1 to June 6, | 69 | 20 | 49 | 0 | 69 |
| Do. from June 6 to Dec. 18, | 115 | 32 | 70 | 13 | 115 |
| Total.... | 184 | 52 | 119 | 13 | 184 |

It must be satifactory to the governors to know that the proportion of deaths during the present year (28 in 100,) is greatly below that of the preceding year, and somewhat less than the average of ordinary seasons. It must also be a source of great gratification to them to reflect that, besides administering to the personal comforts of so large a number of persons afflicted with the most dreadful of all diseases, they have removed from the metropolis as many centres of contagion; —and when it is considered with what facility, during the greater part of the year, the contagion of small-pox was communicated, the advantages which society at large have derived from your establishment must surely be acknowledged as great and substantial.

While, on the one hand, your Institution has been labouring, within its walls, to diminish the evils of this dreadful plague, it has, on the other, been no less assidulously employed in extending to the public, without its walls, the benefits of vaccination. One thousand eight hundred and twenty-two persons have been vaccinated at the hospital since the last half-yearly court, making a total of 3,346 vaccinated between the 1st of January and the 18th of December of the present year. This gives an average of very nearly twelve in each day of the year; and it further shows that about one-seventh of all the children born in London are vaccinated at this establishment. The progress of this very mild disorder having been regularly watched and recorded in almost every one of these cases, your physician is enabled to ensure to this large number of persons all that reasonable share of protection against the small-pox which our present extended knowledge of vaccination permits us confidently to hold out.

Your physician would again, however, urge the importance of inculcating generally that vaccination is not held up and recommended as a complete and unfailing preventive of the small-pox. That a certain proportion of the vaccinated will take small-pox in after-life, the records of your hospital during the current year afford ample testimony. Fifty-seven of the whole number admitted are ascertained to have undergone vaccination; and, though of these a few were affected severely, by much the larger proportion passed through the small-pox in a remarkably mild form. It will be a sufficient proof of this to state, that, of these fifty-seven persons, forty were discharged in perfect health within fourteen days from the period of their admission.

Your physician cannot close his report without adverting to the most important event which has occurred in the history of your hospital during the last six months, viz. the discontinuance of variolous inoculation within its walls. This important measure was in progress before your present physician was engaged in his duty; and he may, therefore, be permitted to express his firm persuasion that it is one which reflects the highest credit upon the establishment, is warranted by extensive observation and adequate experience, and calculated to impress upon the public a just sense of those liberal views which regulate the governors in their management of the Institution. The confidence of the public in the degree of protection afforded by vaccination, is abundantly evinced by the numbers that apply each day to receive it; and your physician, nowise insensible to the results of the experience of the present year, (results which, to a certain extent, must undoubtedly be a matter of regret,) yet feels persuaded that such confidence is well-founded; and that the discontinuance of variolous inoculation to the poor was not only called for by the dictates of reason and experience, but was strictly in accordance with the feelings of that portion of the public for whose benefit your establishment was originally designed, and is still supported.

GEORGE GREGORY, M.D. *Physician to the Small-Pox and Vaccination Hospital.*

8, *Upper John-street*, *Golden-square;*
*December* 19, 1822.

